# Prognostic Assessment and Management of Liver Cirrhosis

**DOI:** 10.1155/2017/5326898

**Published:** 2017-08-01

**Authors:** Xingshun Qi, Ankur Arora, Shanhong Tang, Andrea Mancuso, Fernando Gomes Romeiro

**Affiliations:** ^1^Department of Gastroenterology, General Hospital of Shenyang Military Area, Shenyang, China; ^2^Worthing Hospital, Western Sussex NHS Foundation Trust, West Sussex, UK; ^3^Department of Gastroenterology, General Hospital of Chengdu Military Area, Chengdu, China; ^4^Medicina Interna 1, ARNAS Civico-Di Cristina-Benfratelli, Piazzale Liotti 4, Palermo, Italy; ^5^Epatologia e Gastroenterologia, Ospedale Niguarda Ca' Granda, Piazza Ospedale Maggiore 3, 20162 Milano, Italy; ^6^Faculdade de Medicina de Botucatu, UNESP, Campus de Botucatu, s/n, Botucatu, SP, Brazil

Liver cirrhosis is the 13th leading cause of death worldwide, with increasing mortality rates in the last decades. Many strategies have been developed to reduce the mortality caused by cirrhosis complications, such as ascites, gastroesophageal variceal bleeding, hepatic encephalopathy, and hepatocellular carcinoma. This special issue aimed to provide a platform to explore the prognostic assessment and management of liver cirrhosis. Finally, 12 articles were published, including 4 review articles and 8 research articles. In this editorial, 5 guest editors briefly introduced them.

The deployment of esophageal stent is a novel treatment option for controlling refractory variceal bleeding. In a systematic review with meta-analysis of 5 studies, X.-D. Shao et al. found that nearly all esophageal stents could be successfully deployed with a high rate of complete response, a low incidence of rebleeding, and no stent-related complications. Notably, few patients died of variceal bleeding after the deployment of esophageal stents. Based on the evidence from this study, esophageal stent may be considered for the management of refractory variceal bleeding.

J. M. Choi et al. reported a review on coronary computed tomography angiography in combination with coronary artery calcium scoring for the preoperative cardiac evaluation of liver transplant recipients, discussing the perioperative cardiovascular complications of liver transplantation as a leading cause of postoperative morbidity and mortality following liver transplantation. In fact, ischemic coronary artery disease and cardiomyopathy, the most common cardiovascular diseases, could be negative predictors of postoperative outcomes after liver transplantation. Therefore, comprehensive cardiovascular evaluations are required to assess perioperative risks and prevent concomitant cardiovascular complications after liver transplantation. Coronary artery calcium score (CACS) and coronary computed tomography angiography (CCTA) are the main types of cardiac computed tomography. CCTA in combination with the CACS is a validated noninvasive alternative to coronary angiography for diagnosing and grading the severity of coronary artery disease. A CACS > 400 is associated with significant coronary artery disease and a known important predictor of posttransplant cardiovascular complications in liver transplant recipients. In the review, the usefulness, advantages, and disadvantages of CCTA combined with CACS as a noninvasive diagnostic tool for preoperative cardiac evaluation are discussed.

L. Lei et al. reviewed the recent knowledge regarding diagnostic criteria, treatment, and prognosis of acute kidney injury. Notably, dynamic changes of serum creatinine are still the main diagnostic criteria for acute kidney injury. Other new markers, including CysC (Cystatin c), KIM-1 (kidney injury molecule 1), and NGAL (neutrophil gelatinase associated lipocalin), also have diagnostic value of acute kidney injury.

The risk of fractures in cirrhotic patients is twofold higher than in other people, contributing to the increased mortality observed in these patients. Different from other cirrhosis complications, cirrhosis-related osteoporosis cannot be ameliorated by liver transplantation. In fact, it usually worsens after the use of immunosuppressant drugs. These characteristics have gained space as important concerns among health professionals who treat cirrhotic patients. L. A. A. Santos and F. G. Romeiro presented an extensive review about cirrhosis-related osteoporosis, including definitions related to bone conditions, epidemiological data, pathophysiology, diagnosis, and treatment options. Since cirrhosis-related osteoporosis is a multifactorial disease, the authors used figures to group the most relevant causes according to the bone cells involved, making this part of the text more understandable for the nonspecialist reader. They also consulted more than 130 references to make a deep review on cirrhosis-related osteoporosis, depicting important studies about it. One of the main messages in this article is that cirrhosis-related osteoporosis must be considered in all patients with liver cirrhosis in order to avoid fractures and further complications seen in nontreated cases. The article points out that some treatment options have achieved good results, especially when they are combined. Calcium and vitamin D supplementation is discussed in a practical point of view, as well as the other treatment options. Therefore, the article brings relevant information to improve the care of cirrhotic patients regarding their bone health.

The impact of antiviral therapy on hepatitis C virus infection related liver cirrhosis is unclear. In a retrospective analysis of 25 patients with hepatitis C virus infection related liver cirrhosis, G. Zhang et al. found that the rate of sustained virological response was 72% after the treatment of pegylated interferon plus ribavirin and that sustained virological response was significantly associated with a decrease in the liver fibrosis index and an increase in the albumin levels and platelet counts. However, the authors also emphasized the importance of monitoring the occurrence of hepatocellular carcinoma during a long-term follow-up, considering that the incidence of hepatocellular carcinoma was 16%. Taken together, pegylated interferon plus ribavirin may be helpful for the improvement of prognosis of hepatitis C virus infection related liver cirrhosis.

Shunt stenosis and hepatic encephalopathy are two major complications after transjugular intrahepatic portosystemic shunt. F. He et al. analyzed the impact of pathological features on the development of shunt stenosis and hepatic encephalopathy in 361 patients treated with transjugular intrahepatic portosystemic shunt. The authors provided the novel findings that the risk of shunt stenosis and hepatic encephalopathy were associated with the severity of inflammation and fibrosis in the liver tissue, respectively. Additionally, they found a positive correlation between shunt stenosis and development of hepatic encephalopathy.

It has been well recognized that carvedilol significantly decreases the portal pressure and prevent from variceal bleeding in liver cirrhosis. More recently, several studies also suggest the potential antifibrotic effects of carvedilol in rats intoxicated with CCl4, those with ethanol-induced liver injury, and human umbilical vascular endothelial cell model. X. Tian et al. conducted an experimental study to further explore the antifibrotic effects of carvedilol in a bile duct ligation rat model and its potential mechanisms. The authors found that carvedilol could attenuate the progression of liver fibrosis by reducing the accumulation of collagen and inhibiting the activation of hepatic stellate cells. In addition, its benefit can be gradually strengthened with an increased dose of carvedilol.

Hepatic venous portal gradient is the only established exam for diagnosing portal hypertension. However, it is invasive, expensive, and not widely available. Hence, many studies have been looking for noninvasive markers of portal hypertension using lab tests and indices such as platelet count, prothrombin time, and aspartate aminotransferase platelet ratio, but until now these markers were not deemed as reliably predictors of hepatic venous portal gradient. Thus, other surrogate markers have been searched among imaging exams, such as ultrasonography, computed tomography, and magnetic resonance imaging. When restricted to images, these exams still have limited utility for predicting the presence of portal hypertension. For this reason, new techniques have been developed to predict this finding more accurately, such as magnetic resonance elastography. A. M. Gharib et al. analyzed the usage of magnetic resonance elastography as a surrogate marker of hepatic venous portal gradient measurement in patients with liver disease who were enrolled in a clinical trial to receive simtuzumab, an anti-LOXL2 antibody. The authors found interesting correlations between magnetic resonance elastography, hepatic venous portal gradient, and other variables such as aspartate aminotransferase, gamma glutamyl transferase, soluble LOXL2, liver LOXL2 gene expression, platelets count, and liver biopsy findings. Of note, on stepwise multivariate regression analysis, magnetic resonance elastography was the only variable independently associated with hepatic venous portal gradient, showing that in selected patients this measure can be very useful to estimate the portal pressure.

Nonselective beta blockers (NSBB) constitute one of the mainstays of primary as well as secondary prophylaxis of variceal bleeding in liver cirrhosis. Hepatic venous portal gradient assessment, although an invasive procedure, is currently the only validated method available to monitor the effect of pharmacological therapy in portal hypertension. N. McDonald et al. in their initial small feasibility study have endeavoured to use quantitative MRI markers for the assessment of hemodynamic changes following NSBB usage in portal hypertension. Phase-contrast MR angiography, a noninvasive MR based flow-related technique, was employed to acquire hemodynamic data in selected intra-abdominal vessels at baseline and after 4 weeks of NSBB therapy. Whilst a significant reduction in cardiac output (as measured by superior aortic flow) was documented no significant changes were identified in the visceral blood flow or the T1 relaxation time (in liver and spleen). As the authors rightly concluded larger studies would be required for further consolidating their findings and in determining the true value of noninvasive MR techniques in monitoring hemodynamic changes in portal hypertension.

Hepatocellular carcinoma remains one of the dreaded complications of liver cirrhosis and over the years has evolved as the second most lethal cancer globally. Ultrasound surveillance is an integral part of both the AASLD and EASL guidelines and aims at detecting hepatocellular carcinoma at an early and potentially treatable stage in patient with liver cirrhosis. Early detection and suitable treatment are crucial for improving the disease prognosis, patient survival rates, and their quality of life. But unfortunately early detection of hepatocellular carcinoma may not always be easy because of various reasons including the multifarious appearances the background cirrhotic parenchyma can manifest on ultrasound. M. Soresi et al., following an anecdotal observation of a correlation between coarse nodular pattern of liver parenchyma on ultrasound and evolution into hepatocellular carcinoma, conceived the idea of a longitudinal study investigating the potential risk of hepatocellular carcinoma development in patients with different ultrasound echo patterns. In their prospective study of 359 cirrhotic patients undergoing ultrasound screening the liver echo pattern was assessed and categorized into homogeneous, bright liver, coarse, coarse small nodular pattern, and coarse large nodular pattern. Liver function tests, alpha-fetoprotein assay, and portal hypertension evaluation were other components of the screening program. M. Soresi et al. found alpha-fetoprotein, coarse large nodular pattern, portal hypertension, and age to be independent predictors of hepatocellular carcinoma development. Remarkably, coarse large nodular pattern was found to be a major risk factor for hepatocellular carcinoma wherein 40.7% developed hepatocellular carcinoma. These results are indeed stimulating and could help us diagnose hepatocellular carcinoma early in this subset of patients perhaps by revising the surveillance ultrasound timings. On the contrary, homogeneous and bright liver echo patterns and absence of portal hypertension were not related to development of hepatocellular carcinoma.

Previous studies suggested the potential role of miR-200s (i.e., miR-200a, miR-200b, miR-200c, miR-429, and miR-141) in the development of tissue fibrosis. T. Ma et al. conducted an experimental study to explore the mechanisms of miR-200c in the induction of liver fibrosis. They found that miR-200c activated hepatic stellate cells and then induced liver fibrosis progression via regulating FOG2/PI3K pathway.

M. Liu et al. investigated the role of ERp57 in the development of hepatitis B virus related hepatocellular carcinoma in hepatitis B virus-hepatocellular carcinoma tissues and cell lines, which showed that high expression of ERp57 might lead to poor prognosis in patient with hepatitis B virus related hepatocellular carcinoma. They indicate that ERp57 might be a potential biomarker of these patients. The mechanism and related signal transduction pathways of ERp57 in pathogenesis of hepatitis B virus-hepatocellular carcinoma should be further studied.

*Xingshun Qi* *Ankur Arora* *Shanhong Tang* *Andrea Mancuso* *Fernando Gomes Romeiro*

